# iMEGES: integrated mental-disorder GEnome score by deep neural network for prioritizing the susceptibility genes for mental disorders in personal genomes

**DOI:** 10.1186/s12859-018-2469-7

**Published:** 2018-12-28

**Authors:** Atlas Khan, Qian Liu, Kai Wang

**Affiliations:** 1 Division of Nephrology, Department of Medicine, College of Physicians and Surgeons, Columbia University, New York, NY 10032 USA; 2 Raymond G. Perelman Center for Cellular and Molecular Therapeutics, Children’s Hospital of Philadelphia, Philadelphia, PA 19104 USA; 3 Department of Pathology and Laboratory Medicine, University of Pennsylvania Perelman School of Medicine, Philadelphia, PA 19104 USA

**Keywords:** Structural variants (SVs), Single nucleotide variants (SNVs), Machine learning, Deep neural network, Mental disorders, Personal genome

## Abstract

**Background:**

A range of rare and common genetic variants have been discovered to be potentially associated with mental diseases, but many more have not been uncovered. Powerful integrative methods are needed to systematically prioritize both variants and genes that confer susceptibility to mental diseases in personal genomes of individual patients and to facilitate the development of personalized treatment or therapeutic approaches.

**Methods:**

Leveraging deep neural network on the TensorFlow framework, we developed a computational tool, integrated Mental-disorder GEnome Score (iMEGES), for analyzing whole genome/exome sequencing data on personal genomes. iMEGES takes as input genetic mutations and phenotypic information from a patient with mental disorders, and outputs the rank of whole genome susceptibility variants and the prioritized disease-specific genes for mental disorders by integrating contributions from coding and non-coding variants, structural variants (SVs), known brain expression quantitative trait loci (eQTLs), and epigenetic information from PsychENCODE.

**Results:**

iMEGES was evaluated on multiple datasets of mental disorders, and it achieved improved performance than competing approaches when large training dataset is available.

**Conclusion:**

iMEGES can be used in population studies to help the prioritization of novel genes or variants that might be associated with the susceptibility to mental disorders, and also on individual patients to help the identification of genes or variants related to mental diseases.

**Electronic supplementary material:**

The online version of this article (10.1186/s12859-018-2469-7) contains supplementary material, which is available to authorized users.

## Background

Mental disorders, such as schizophrenia, bipolar disorder, attention-deficit/hyperactivity disorder (ADHD), autism spectrum disorder (ASD), major depressive disorder (MDD) and language/communicative impairments, have been found to affect ~ 25% people worldwide at some point in their lives [1]. Thus, mental disorders have been placed as one of the leading causes of disability and they take a significant social and economic toll to the society. Genetic factors have been suggested to be a strong contributors to neuropsychiatric and neurodevelopmental disorders by a wide range of evidence in existing work [2–9]. Hundreds of variants with small effect sizes have been identified by standard genome-wide association studies (GWAS) in several mental disorders [10–13], and a number of rare CNVs associated with a range of mental disorders have also been detected by genome-wide copy number variations (CNVs) studies [14–17]. Recently, de novo mutations in specific genes or pathways were also found to be associated with several mental disorders [18–20]. With the recent rapid development of next-generation sequencing techniques, a large amount of high-throughput whole-genome sequence data were generated, making it possible to study well-phenotyped patients with mental disorders by examining all types of genetic variations in their genomes. Improved understanding of the genetic basis of mental disorders could be obtained by direct identification of casual variants rather than proxy markers from whole-genome genotyping.

However, there are still at least three critical problems in genetic analysis of mental disorders. First, although by using high-throughput genomic data, a range of rare and common genetic variants have been discovered to be potentially associated with mental disorders with varying effect sizes [21–24], many more have not been uncovered. Second, there are also a lack of powerful integrative methods to systematically prioritize variants and genes that confer susceptibility to mental disorders. Third, with the discovery of candidate variants for mental diseases, it is not easy to design appropriate functional follow-up experiments since the mechanisms of action are still unclear based on those candidate variants. With these unsolved problems, a large gap clearly exists between the amount of data for genetic variations and the comprehension on how they impact diseases, resulting in a substantial delay to develop targeted treatment approaches. Many computational algorithms have been proposed to study coding variants which might affect protein functions, but how non-coding variants impact mental diseases is very challenging. With increasing volume of genomic data, a lot of functional DNA elements in human genome have been identified [25], and different computational tools and various machine learning algorithms have been designed to distinguish pathogenic and neutral variants for both coding and non-coding mutations, such as the CADD score (Combined Annotation Dependent Depletion) [26], the DANN score [27], the GWAVA score [28], the FATHMM-MKL score [29], the deltaSVM score [30], the DeepSEA (Deep learning–based SEquence Analyzer) score [31], as well as other similar scores such as the GTEx (Genotype-Tissue Expression) score [32] and the intolerance score [33]. Many sets of available annotations enabled the study of how coding and non-coding variants function in mental diseases, and we previously developed a method for variant prioritization by integrating various computational functional methods for non-coding variants scores for mental disorders [34]. However, there are still a lack of tools available specifically to predict the consequences of non-coding variants for mental disorders on personal genomes that consider the specific properties of mental diseases and neuronal genes. To extend our previous work [34], we developed a novel bioinformatics tool, integrated MEntal-disorder GEnome Score (iMEGES), which leverages a two-steps strategy to predict the impacts of variants and genes in personal genomes on mental diseases:

- In the first step of iMEGES, we used a deep learning approach to build a whole genome variant score for variants which affect brain functions, and to prioritize non-coding variants and to generate non-coding variants score for brain disorders called ncDeepBrain score.
- In the second step of iMEGES, we used another deep learning framework to integrate the ncDeepBrain score, general gene scores (such as GTEx), and disease-specific scores to prioritize mutated genes for mental disorders based on individual patient’s own phenotype and genotype information.

iMEGES was evaluated on a few publicly available data sets of mental disorders. We believe that iMEGES can be used in population studies to prioritize novel genes or variants which might be associated with the susceptibility of mental diseases, and also on individual patients to help identify genes or variants related to mental diseases. iMEGES is available at https://github.com/WGLab/iMEGES.

## Methods

### Schematic framework of iMEGES

**Fig. 1:**
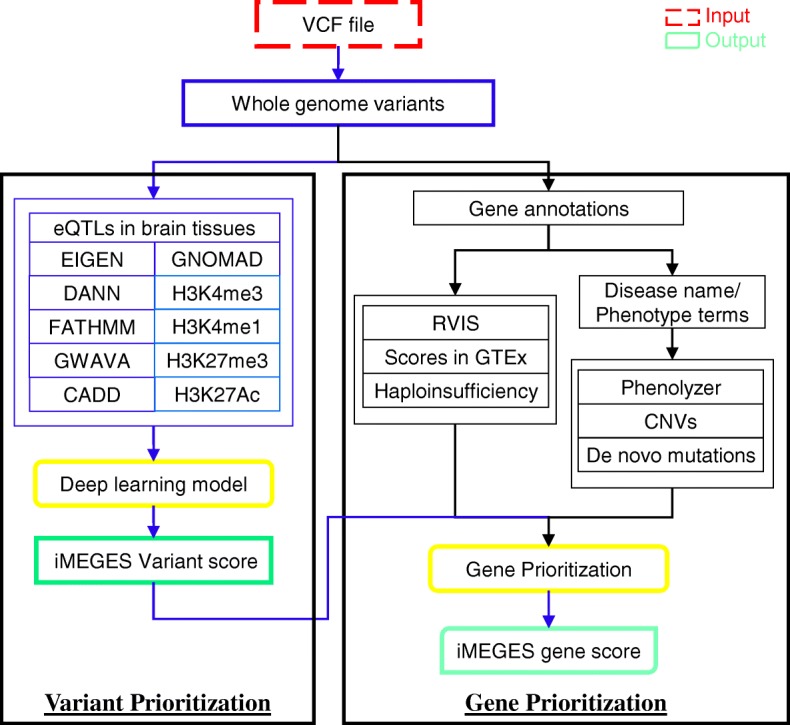
Schematic overview of iMEGES. The input are whole genome variants from patients with mental disorders, in ANNOVAR input format, BED format or VCF format. In variant prioritization of iMEGES, iMEGES extracts predicted scores from various predictors for non-coding variants, the GNOMAD frequency, known brain eQTLs scores, enhancer and promoter regions of the brain, and then trains a deep learning algorithm for generating variant score named as ncDeepBrain. In gene prioritization, iMEGES integrates the variant score from the first step, general gene scores (RVIS, GTEx and haploinsufficiency scores), and disease-specific scores such as Phenolyzer, CNVs and de novo mutations scores to generate iMEGES gene prioritization score for mental disorders

As shown in Fig. 1, the input to iMEGES is genetic mutations and phenotypic information from a patient. The input format could be ANNOVAR [35] input format, VCF format, or BED format. The outputs of iMEGES are the ranking of whole genome susceptibility variants together with the detailed information for each variant, and the prioritized disease-specific genes together with iMEGES scores for mental disorders. Correspondingly, iMEGES contains two main steps (see Fig. 1), variant prioritization and gene prioritization.

For non-coding variants (whole genome variants), variant scoring step in iMEGES generates non-coding variants score from various existing prediction algorithms, and then prioritizes susceptibility variants for mental disorders. Several non-coding scores from existing predictors were used to prioritize non-coding variants, such as the known eQTLs data from CommonMind project in brain tissues, and enhancers/promoters regions from PsychENOCDE and RoadMap Epigenomics projects. These scores were integrated by a deep learning process in our model to generate our variants score named as the ncDeepBrain score. The ncDeepBrain score supplements functional scores for coding variants and structural variants in the genome. Gene prioritization in iMEGES uses a deep learning framework and takes as input various variables: the ncDeepBrain scores of the first step, general gene-specific scores (such as RVIS, GTEx and haploinsufficiency scores), and disease-specific scores (such as Phenolyzer, CNVs and de novo mutations scores) to prioritize genes for mental diseases. The details of the two steps are described below.

### Variant prioritization

Variant prioritization at iMEGES integrates various non-coding scores, the known eQTLs, and enhancers/promoters regions for ranking variants. These scores are described below.

#### Non-coding scores

Non-coding scores used in iMEGES includes EIGEN [36], CADD [26], DANN [27], GWAVA [28] and FATHMM (Functional Analysis through Hidden Markov Models) [29]. The EIGEN score measures functional importance and is generated by unsupervised machine learning based on diverse annotations [36]. The EIGEN score is publicly available for 9 billion variants. The CADD was generated by support vector machine (SVM) to discriminate observed variants 14.7 million high-frequency from simulated 14.7 million variants [26]. Based on the same data used in training of CADD scores, a deep learning approach called DANN was also developed to discriminate observed variants from simulated variants [27]. GWAVA is whole genome score based on modified random forest algorithm [28]. In GWAVA, 174 different genomic and epigenomic annotations were used to define a new whole genome variants GWAVA score. The FATHMM score can be used to estimate the impact of both coding and non-coding variants [29]. In iMEGES, ANNOVAR [35] was used to extract all ~ 9 billion possible SNPs in the human reference genome (GRCh37), and genome-wide pre-computed prediction scores predicted by these computational tools were used for variant prioritization.

#### Enhancer and promoter regions

The ChIP-seq data used in variant prioritization includes EpiMap (doi:10.7303/syn4566010), CNON (doi:10.7303/syn4590897) and Yale-ASD (doi:10.7303/syn4566141) for different types of brain tissues, which were downloaded from the PsychENCODE project [37] (Please refer to our work [34] for detail.). Variants based on the ChIP-seq data from these projects were then annotated using ANNOAR [35] to know whether variants are likely functional by inspecting where the mutations are located, within or outside the ChIP-seq region.

The enhancer and promoter data of brain from Roadmap Epigenomics Project (http://www.roadmapepigenomics.org) was also used in variant prioritization. This data set contains various brain tissues such as angular gyrus, anterior caudate, cerebellum, cingulate gyrus, fetal, hippocampus middle, inferior temporal lobe, mid frontal lobe and substantia nigra. Existing works have found that significant gene expression changes, resulting from the variation in regulatory regions such as SNVs and CNVs, might be directly link to pathogenicity [38, 39], and are more common in mental disorder such as schizophrenia and ASD [40, 41]. Therefore, non-coding variants in the enhancer and promoter regions of brain were also integrated into variant prioritization according to Eq. (1).

1 $$ f\left(\mathrm{variant}\right)=\left\{\begin{array}{c}1,\mathrm{if}\ \mathrm{a}\ \mathrm{variant}\in \left(\mathrm{the}\ \mathrm{regions}\ \mathrm{of}\ \mathrm{enhancers}\ \mathrm{and}\ \mathrm{promoters}\right)\\ {}0,\mathrm{if}\ \mathrm{a}\ \mathrm{variant}\notin \left(\mathrm{the}\ \mathrm{regions}\ \mathrm{of}\ \mathrm{enhancers}\ \mathrm{and}\ \mathrm{promoters}\right)\end{array}\right. $$

#### Expression quantitative trait locus (eQTLs)

Expression Quantitative Trait Loci (eQTLs) are genomic loci whose variants are closely associated with the changes in gene expression and can be potential loci for brain disorders [42]. Existing studies have examined such SNP-transcript association in different brain samples [42–44]. In this study, significant eQTLs were downloaded from [44] and the CommonMind database [45] where the 6.4 million genotyped and imputed markers with estimated allele frequency ≥ 0.05 and 16,423 genes were generated for analysis in variant prioritization based on Eq. (2).

2 $$ f(variant)=\Big\{{\displaystyle \begin{array}{l}0.5, if\ a\  variant\ does\ not\ have\ a\  known\ brain\ eQTLs\ score\\ {} score, if\ a\  variant\  has\  known\ brain\ eQTLs\ score\end{array}}\operatorname{} $$

In iMEGES, all the variants were annotated using ANNOVAR [35], and different variant scores, including EIGEN [36], CADD [26], DANN [27], GWAVA [28], FATHMM [29], GNOMAD frequency [46], the known brain eQTLs from CommonMind, enhancers/promoters data from PsychENOCDE and Roadmap Epigenomics projects were then used to prioritize coding and non-coding variants for mental diseases.

### Gene prioritization

Gene prioritization in iMEGES takes the input of the ncDeepBrain score of the first step, RVIS, GTEx, haploinsufficiency scores, and disease-specific scores (such as Phenolyzer, CNVs and de novo mutations in mental disorders). To link the ncDeepBrain scores of non-coding variants to genes, we used a genomic distance (≤ 100 kb) between SNP marker and gene position. Given the fact that some genes harbor more than one mutation, we used all the mutations and prioritized each of the variants for specific mental disorders genes. Other scores used in gene prioritization were calculated by existing tools and details are given below.

### General scores

#### Genotype-tissue expression (GTEx) score

Recently thousands of loci have been detected by GWAS for common diseases [10, 47–49] and hundreds of susceptibility genes were identified for many human conditions and quantitative traits [50, 51]. However, for most of the loci, the mechanisms underlying disease susceptibility remain unknown. The Genotype-Tissue Expression (GTEx) project was developed and a database was provided to scientific community [32], to study the association between genetic variations and gene expression in 44 different human tissues. We downloaded the GTEx scores for all tissues, using a stringent threshold of Q-values < 0.05 for defining significant associations. The GTEx score for each of the SNPs-genes pairs was used in iMEGES.

3 $$ f(variant)=\Big\{{\displaystyle \begin{array}{l} EMR, if\ a\  variant\ does\ not\ have\ a\  GTEx\ score\\ {} score, if\ a\  variant\  has\  GTEx\ score\ q- value\end{array}}\operatorname{} $$

where EMR is the estimate of missing rate of GTEx score described later.

#### Residual variation intolerance (RVIS) score

The RVIS score measures the tolerance of genes to mutations. We downloaded RVIS scores from [33] for gene prioritization by using Eq. (4).

4 $$ f(gene)=\Big\{{\displaystyle \begin{array}{l}0, if\ a\  gene\ does\ not\ have\ a\  RVIS\ score\\ {} score, if\ a\  gene\  has\  RVIS\ score\end{array}}\operatorname{} $$

#### Haploinsufficiency score

Haploinsufficiency refers to the biological insufficiency of a single functional copy of a gene to maintain the normal function which might cause many dominant diseases [52]. We downloaded the haploinsufficiency score from [53] and used these scores in gene prioritization in iMEGES.

### Disease specific scores

#### Phenolyzer score

Phenolyzer is a computational tool, which prioritizes disease genes based on a list of phenotype terms, and can facilitate the analysis of whole genome and exome sequencing studies [53]. Phenolyzer score can be used for each of the mental disorder diseases: schizophrenia, ASD, ADHD and MDD. However, when more detailed phenotype information for each patient is available (such as Human Phenotype Ontology terms), they can be optionally used as input to Phenolyzer to obtain prioritized gene scores. For whole genome variants, we used ANNOVAR to annotate variants and each non-coding variant was assigned to its closest gene based on genomic distance.

5 $$ f(gene)=\Big\{{\displaystyle \begin{array}{l} EMR, if\ a\  gene\ does\ not\ have\ a\  Phenolyzer\ score\\ {} score, if\ a\  gene\  has\  Phenolyzer\ \mathrm{s} core\end{array}}\operatorname{} $$

where EMR is the estimate of missing rate for several diseases such as schizophrenia and autism.

#### Copy number variations (CNV) score

Copy number variations (CNV) are traditionally defined as duplication or deletions of genome fragment more than 1 kb, when compared to the human reference genome. Previous studies demonstrated that CNV may account for a significant proportion of human genome even when analyzing healthy subjects [54]. The genome-wide association studies (GWAS) on mental disorders can also analyze CNV sites using the SNP genotyping data. Marshall et al. studied schizophrenia cohort of 21,094 cases and 20,227 controls to investigate the contribution of CNVs to etiology of schizophrenia [55]. We downloaded the CNV data of schizophrenia from [55], which contains 1309 genes with significant q-values. We used q-values for significant genes in gene prioritization based on Eq. (6):

6 $$ f(gene)=\left\{\begin{array}{c}0.5, if\ a\  gene\ does\ not\ have\ a\  CNV\  score\ \\ {}\  score, if\ a\  gene\  has\  CNV\  score\ \left(q- value\right)\ \end{array}\right. $$

#### De novo mutations (DNM) score

Each individual person may carry some new variants, which are not present in the genomes of their parents and thus denoted as de novo mutations. Most of de novo mutations do not cause diseases [56] or they may merely represent false variant calls, however, some de novo mutations may contribute to different types of disease/phenotype [57, 58]. Several severe developmental/mental disorders, such as autism [17, 59, 60] and schizophrenia [18, 61], were found to have enrichment in damaging de novo mutations in developmentally important genes. We downloaded whole genome de novo mutations from de novo mutations database for neuropsychiatric disorders (http://www.wzgenomics.cn/NPdenovo/download.php), and de novo mutations from db-denovo database (http://denovo-db.gs.washington.edu/) of different published studies [62]. We used all these de novo mutations in our feature vector of gene prioritization to prioritize human genes involved in human brain disorders.

For a quick reference, the scores and tools used were summarized in Additional file 1: Table S1.

### Deep neural network model in iMEGES

For both variant prioritization and gene prioritization in iMEGES, corresponding features above would be used as input of deep learning framework, and there were two similar deep learning frameworks: one for variant prioritization and the other for gene prioritization. The details are described below.

Deep learning framework used in this study is a typical multilayer neural network with one input layer, one output layer and several hidden layers. Each of hidden layers consists of several computational neurons, and several sequential layers are organized to conduct sequential functional transformations. A neuron in a layer is fed by input data or the output from a set of previous-layer neurons and generated a single value as output.

In iMEGES, four hidden layers were used, and the number of hidden nodes was tuned. To void diminishing effect in neural network, we applied the well-known dropout strategy where a proportion of neurons at a specific layer are randomly set to a value of 0 in each step to regularize the model. The dropout rates of neurons need to be manually tuned to generate better model. For the last layer of each step in iMEGES, the sigmoid output layer ($\widehat{\mathrm{y}}=\mathrm{Sigmoid}\left(\mathrm{X}\right)=\frac{1}{1+{e}^{-\left[{W}^TX+b\right]}}$) was used to make predictions for variants/genes and the output scores were scaled to the 0–1 range. Here, X denotes input matrix, W representes weight matrix for the sigmoid output layer, T refers to transpose, b is bias term in linear combination of predictors, and $\widehat{\mathrm{y}}$ is final sigmoid function. For variant prioritization, the used features X are

$$ F=\left(\begin{array}{l}\mathrm{EIGEN},\mathrm{CADD},\mathrm{DANN},\mathrm{GWAVA},\mathrm{FATHMM},\mathrm{GNOMAD},\\ {}\mathrm{eQTLs},\mathrm{H}3\mathrm{K}4\mathrm{me}3,\mathrm{H}3\mathrm{K}4\mathrm{me}1,\mathrm{H}3\mathrm{K}27\mathrm{me}3,\mathrm{H}3\mathrm{K}27\mathrm{Ac}\end{array}\right) $$

For gene prioritization, the used features X are

$$ \mathrm{F}=\left(\mathrm{ncDeepBrain},\mathrm{RVIS},\mathrm{GETx},\mathrm{Haploinsufficiency},\mathrm{Phenolyzer},\mathrm{CNV},\mathrm{de}\ \mathrm{novo}\ \mathrm{mutation}\right). $$

The objective function to be minimized in iMEGES is the sum of the negative log likelihood $L\left(y,\widehat{y}\right)=- ylog\widehat{y}-\left(1-y\right)\log \left(1-\widehat{y}\right)$ where y are actual labels in training datasets while ŷ are prediction. This objective function is optimized according to the stochastic gradient descent with momentum using standard back-propagation algorithm. As for mutations with missing values, we used bPCA (Bayesian Principal Component Analysis) fill to impute values for each missing value of variants. bPCA is a computational tool to estimate missing values in large dataset [63]. The imputation was conducted for DANN, CADD, EIGEN, GWAVA and FATHMM using non-missing scores of each of variants for all potential SNVs in human whole genome. The missing values were summarized in Additional file 1: Table S2.

Our model implemenation utilized the Keras library (https://keras.io/) with TensorFlow as a backend (https://www.tensorflow.org/) for deep learning in iMEGES to rank the variants and genes for brain. The correlation between all the feature scores was also investigated in our training dataset to build efficient deep learning model with proper feature scores.

A ten-fold cross-validation was used to test predictive performance of iMEGES with estimated receiver operating characteristic (ROC) curve with sensitivity against specificity and area under curve (AUC), where the ROC plots were generated using python scikit, a machine learning library in python. Imbalanced data refers to a problem when one of the classes is rare over the other class. We used ROSE R library to handle imbalanced testing and training datasets for iMEGES [64].

### Datasets used in iMEGES

Four training and four testing datasets were used for variant prioritization in iMEGES.

#### Training dataset 1: DNase I sensitivity quantitative trait loci (dsQTL) data

The first training data were downloaded from [30], including 574 dsQTL positive SNPs and 27,735 negative SNPs with minor allele frequency (MAF) > 5% in dsQTL regions. dsQTL positive SNPs were strictly selected by deltaSVM [30] to ensure the causality of dsQTL SNPs to DNase I sensitivity change.

#### Training dataset 2: GWAVA-region

In the second training data, there are 1614 non-coding regulatory SNPs downloaded from HGMD (human gene mutation database with the April 2012 release using GWAVA) [28], while negative common SNVs were randomly selected from variants with MAF > 1% from the 1000 genomes project [65].

#### Training dataset 3: Expression quantitative trait loci fine-mapping data

The third training dataset contains 31,118 functional eQTLs which were generated from joint test of 7 brain tissues/cell lines from eleven studies [66, 67], and an equal number (36,540) frequency-matched background SNPs which were sampled around the nearest TSS of randomly selected genes.

#### Training dataset 4: Expression quantitative trait loci data

The fourth training dataset used in this study was generated by DeepSEA [31]. In this dataset, the associated SNPs were generated with *P*-value cutoff 1 × 10^− 10^ from the non-coding eQTLs of GRASP (Genome-Wide Repository of Associations between SNPs and Phenotypes) [68], and the non-associated SNPs were generated from 1000 Genomes Project [69], and randomly selected from those SNPs which are closest to associated SNPs and with matched minor allele frequency distribution from associated SNPs.

#### Testing dataset 1: Schizophrenia

The first testing dataset is for schizophrenia, and has 3440 significant SNPs and 66,916 non-significant SNPs [70], and downloaded from PGC (Psychiatric Genomics Consortium: https://www.med.unc.edu/pgc). In this study, the significant positive SNPs must have P-value less than 1 × 10^− 10^ and non-significant negative SNPs have P-value > 0.5. Positive and negative SNPs must have matched frequency.

#### Testing dataset 2: Autism spectrum disorder

The second testing dataset was downloaded from [71] for ASD. In this dataset, the 8002 significant SNPs have *P*-value not higher than 0.0227, and the 19,322 non-significant SNPs have P-value not less than 0.06.

#### Testing dataset 3: Regulatory variants

The third testing dataset was downloaded from [66] with manual curation. This dataset contains 76 regulatory variants which were experimentally validated, and 156 background SNPs which were frequency-matched from nearby regions of regulatory variants.

#### Testing dataset 4: Synonymous pathogenic variants

The fourth testing dataset has 477 de novo synonymous variants compiled by Gelfman et al. [72]. Using the pathogenicity (TRaP) score designed in [72], 75 of 477 variants are determined as pathogenic, and the rest 402 variants are not-associated.

## Results

iMEGES has two deep learning modules, one for variant prioritization and the other for gene prioritization for mental disorders. Variant prioritization prioritizes the susceptibility variants according to the ncDeepBrain score which was generated by integrating scores from various predictors for non-coding variants, the known eQTLs from CommonMind project in brain tissues, and enhancer/promoter regions from the PsychENOCDE and RoadMap Epigenomics projects. After that, another deep learning framework takes as input the ncDeepBrain score, three gene-based scores of RVIS, GTEx and haploinsufficiency scores, and three disease-specific scores from Phenolyzer, CNVs and de novo mutations on mental disorders, to prioritize mental disease genes. Below, we detailed the performance for each of the two modules of iMEGES and demonstrated the performance of iMEGES in real-world applications.

### Variant prioritization

In variant prioritization (ncDeepBrain) of iMEGES, both tissue-related scores and other general (non-tissue-specific) scores for non-coding variants were used. General scores, such as these scores from EIGEN, CADD, DANN, GWAVA and FATHMM, provide general information of non-coding variants at a genomic scale. Tissue-related scores of known eQTLs in brains from CommonMind, and enhancer/promoter from the PsychENCODE project might contain more specific information related to brain tissues and mental disorders. The two types of scores were integrated by a deep learning framework (ncDeepBrain for short in iMEGES).

**Fig. 2:**
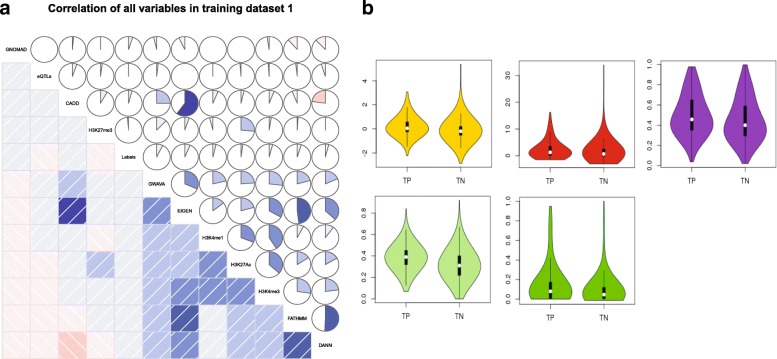
Correlation between the predictors used in iMEGES on training dataset 1. The feature vector contains different non-coding variants scores, enhancers, promoters and known brain eQTLs for variant prioritization of iMEGES. **a** Correlation plots illustrate Pearson correlation between each of all scores from the predictors and outcome on the training dataset. The level of correlation was indicated by color and size of the shaded regions in the pie charts at upper right, and blue and larger proportions of the shaded regions suggest higher positive correlation. **b** Violin plots of scores from various predictors on the training datasets in associated and non-associated groups, and in each violin plot, the median and the first through third interquartile range of predicted scores in each group were provided

**Fig. 3:**
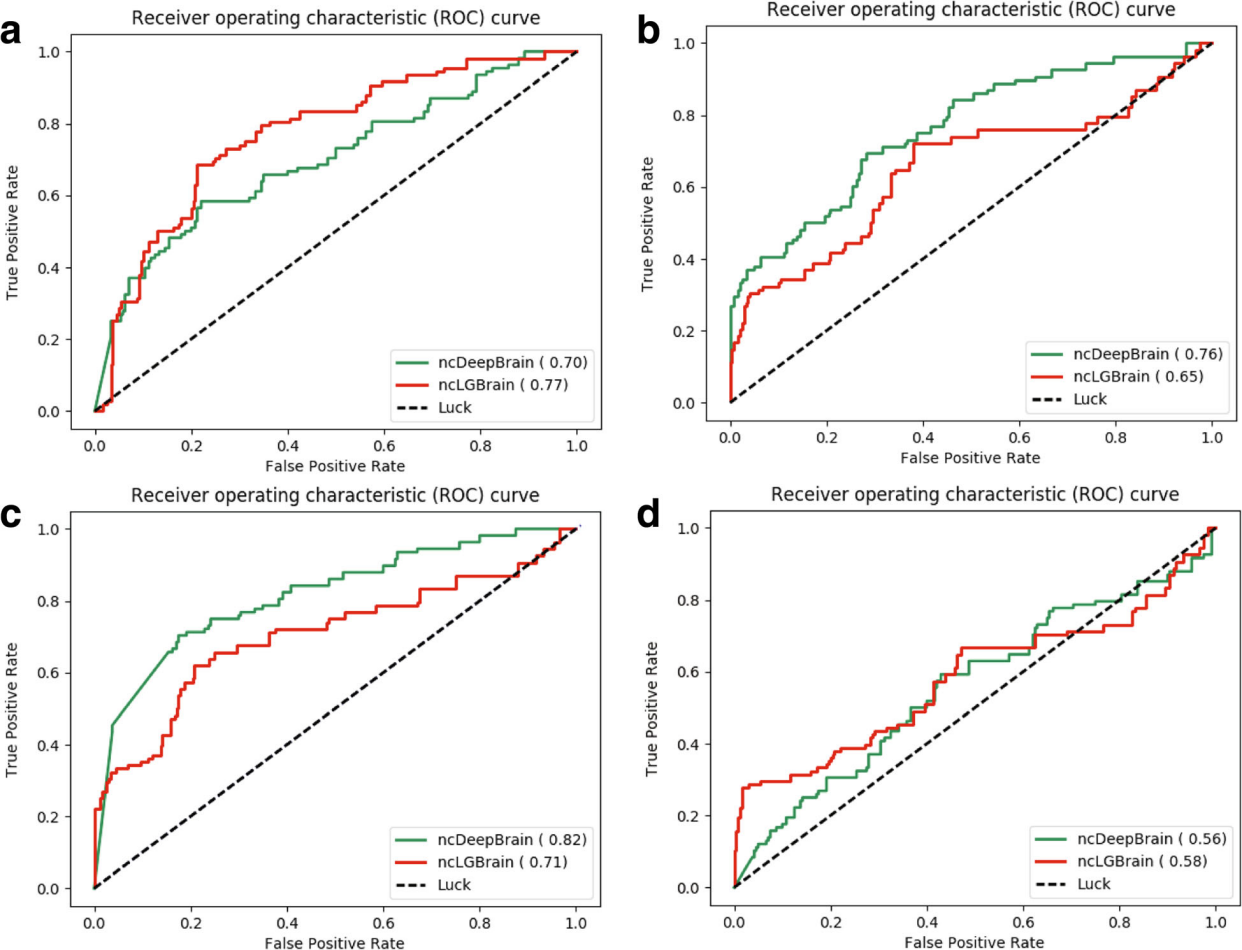
The AUC performance of non-coding variant prioritization of iMEGES for ncDeepBrain (a deep learning model) and ncLGBrain (logistic regression) scores. The higher AUC score is, the better performance is for discriminating disease-related variants from neutral variants. The performance values were achieved by ncDeepBrain and ncLGBrain on testing dataset 3, when ncDeepBrain and ncLGBrain were trained on training sets 1, 2, 3 and 4 respectively for (**a**), (**b**), (**c**) and (**d**)

To test the redundancy of different scores of genetic variants conferring susceptibility to mental diseases [73], Pearson’s correlation coefficient of each pair of these scores for non-coding variants were calculated and presented in Fig. 2(a). Violin plots of all predictors on training dataset 1 were also shown in Fig. 2(b) to confirm that the distributions of these scores for associated and non-associated variants do not contains outliers and the correlation of the variables in the training dataset 1 is tabulated in Additional file 1: Table S3. To compare the performance of variant prioritization for ncDeepBrain, we generated ROC curves for discriminating disease variants from non-disease variants (normal) in the testing set and calculated AUC scores. The classification AUC value of ncDeepBrain is 80% on the deltaSVM data training dataset 1 as shown in Additional file 1: Figure S2(a). We also trained ncDeepBrain on the GWAVA data of positive and negative SNPs. The classification AUC value of ncDeepBrain on the GWAVA’s paper data is 91% (see Additional file 1: Figure S2(c)). ncDeepBrain works well on this data (see Fig. 3(c)). However, GWAVA was trained using Human Gene Mutation Database (HGMD) and may suffer from overfitting issues [74]. The AUC values of ncDeepBrain on testing dataset 1 and testing dataset 3 are 75% and 89% respectively (Additional file 1: Figure S3(a, c)).

The classification AUC value of ncDeepBrain on PGC schizophrenia data is 75%, and ncDeepBrain works better on this data, however its AUC value is still unsatisfactory on testing dataset 1 as shown in Additional file 1: Figure S1 (b). We suspect that it might be due to the fact that TN (true negative) and TP (true positive) variants are not well defined, since the variants were sampled based on imputed *p*-values/variants frequency from genome-wide association studies (GWAS), and most of these variants may be proxy markers of causal variants.

We also tested ncDeepBrain and logistic regression ncLGBrain models on testing datasets in Table 1 for discriminating disease variants from neutral variants. For training dataset 1, 4 and testing dataset 3, ncLGBrain performed better than ncDeepBrain (see Fig. 3(a, d)), however for training 2, 3 datasets ncDeepBrain performed better than ncLGBrain (see Fig. 3 (b) and (c)).

**Table 1:** The training and testing datasets variant prioritization of iMEGES

Dataset	Positive	Negative	Description
Training dataset 1	574	27,735	The most likely causal dsQTL SNPs were downloaded from deltaSVM [30]
Training dataset 2	1614	161,400	Regulatory associated mutations were downloaded from HGMD from 2012, and random SNVs with allele frequency ≥ 1% in the 1000 Genomes Project
Training dataset 3	31,118	36,540	eQTLs SNPs were collected from 11 studies on 7 tissues/cell lines
Training dataset 4	78,613	593,335	Non-coding eQTLs from GRASP was considered to be associated, while SNPs from 1000 Genomes Project not to be associated
Testing dataset 1	3439	66,916	Based on P-values of imputed SNPs from Psychiatric Genome Consortium (PGC) schizophrenia GWAS
Testing dataset 2	8002	19,322	Based on P-values of imputed SNPs from Psychiatric Genome Consortium (PGC) autism spectrum disorder (ASD)
Testing dataset 3	76	156	Manually curated regulatory SNPs with experimental validation.
Testing dataset 4	75	402	The synonymous variants compiled by [72]

Additionally, we trained both models on training datasets 1, 2, 3, 4 and tested on testing dataset 3. For training dataset 1 and testing dataset 1, ncDeepBrain outperforms the logistics regression as shown in Additional file 1: Figure S1(a), where the AUC value of ncDeepBrain is 61% and the AUC value of ncLGBrain is 55%, for training dataset 2 and testing dataset 1, the ncLGBrain outperformed the ncDeepBrain (see Additional file 1: Figure S1 (b)). For training dataset 2, 3 and testing dataset 1 both the models performed quite similar (Additional file 1: Figure S1 (b, c)). For training dataset 4 and testing dataset 1, ncDeepBrain outperformed the ncLGBrain model (see Additional file 1: Figure S1(d)). The AUC value of ncDeepBrain is low for training datasets 1, 2, 3, 4 and testing dataset 2 (as shown in Additional file 1: Figure S4), possibly due to the false positives and false negatives in the testing dataset 1.

Furthermore, we compared our ncDeepBrain score with each of the individual scores such as EIGEN, CADD, DANN, GWAVA and FATHMM. The ncDeepBrain outperformed the existing methods in terms of AUC value for training dataset 1 (see Additional file 1: Figure S5). For training dataset 1, the AUC values of EIGEN, CADD, DANN, GWAVA and FATHMM are 52%, 57%, 57%, 59% and 57% respectively (Additional file 1: Figure S5), but the AUC value achieved by ncDeepBrain is 80%, which substantially outperformed each individual score (See Additional file 1: Figure S2(a)).

To evaluate the approach for the prioritization of disease-relevant variants for personal genomes in individuals affected with mental disorders, we analyzed the whole-genome sequencing data on two patients affected with autism spectrum disorders, which were previously published [75]. In the original publication published five years ago, we detected 59 candidate coding variants which might increase susceptibility to autism, and further identified ANK3 as the most likely candidate gene by manual examination. In [75], we also identified 33 prioritized non-coding variants with evolutionary constraint and experimental evidence from ENCODE. We hypothesize that additional annotation information such as PsychENCODE and Roadmap Epigenome Project that are available today can help us further refine possible disease-relevant variants. Therefore, we re-analyzed the previously published data set, and found that 19 of 33 variants are in PsychENCODE peaks or Epigenome peaks.

### Gene prioritization

Gene prioritization of iMEGES used a deep learning framework to integrate the ncDeepBrain score, general scores (such as GTEx score of the variants with q-value less than or equal to 0.05 [32] for each of the 44 available tissues from the GTEx database, RVIS gene score [33], a haploinsufficiency score and the disease specific scores (such as Phenolyzer score for each gene [53], CNVs and de novo mutations scores of mental disorders). The purpose of this step is to discriminate causal genes from genes unrelated to mental disorders, and then to generate the iMEGES score to prioritize susceptibility genes which might be associated with mental diseases. For a given patient, it is also possible to prioritize the disease-related genes based on genomic profile of this patient by integrating variant-level and gene-level scores.

First we examined the summary statistics for all feature variables to ensure validity of these variables used in gene prioritization. We investigated whether there were any outliers which are biologically feasible, and used pairwise correlation to diagnose the collinearity of the variables to make ensure that no feature variables are collinear.

**Fig. 4:**
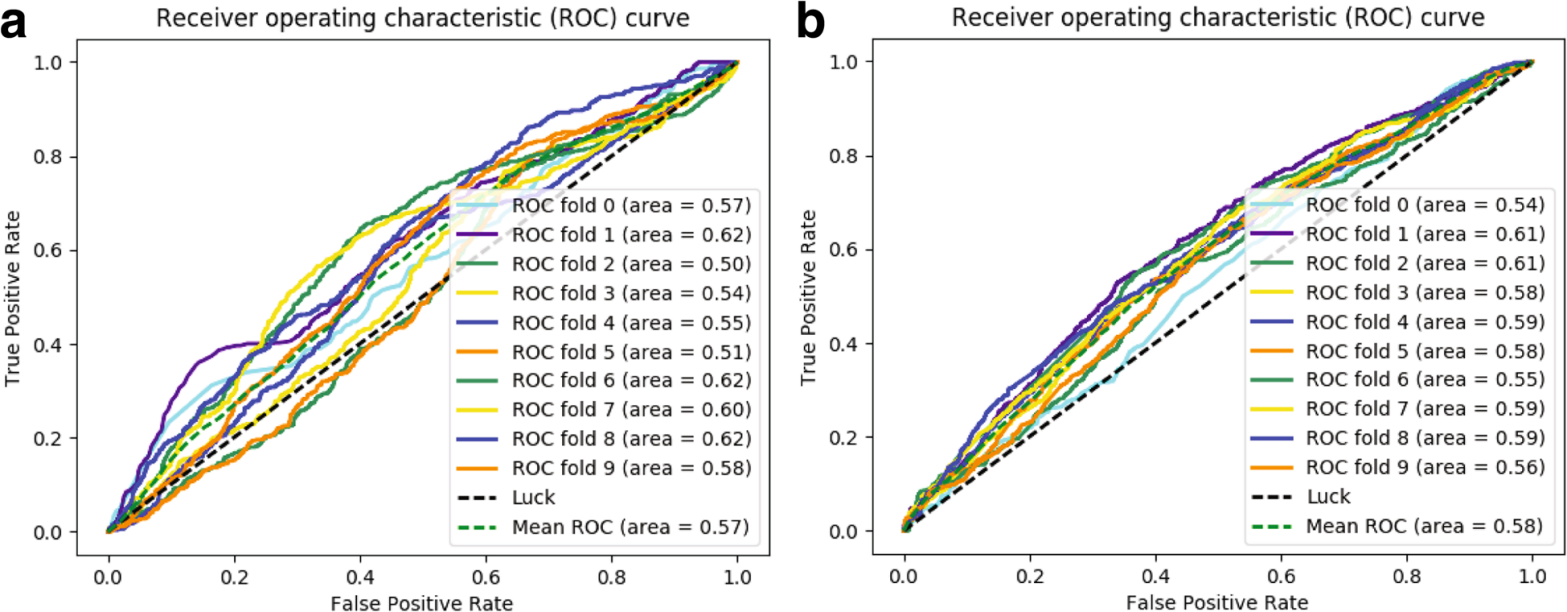
The performance of gene prioritization of iMEGES. **a** the performance of gene prioritization of iMEGES on the schizophrenia data; **b** the performance of gene prioritization of iMEGES on the ASD data

We evaluated the performance of gene prioritization in iMEGES on the schizophrenia dataset. The performance of iMEGES for gene prioritization are shown in Fig. 4. Here the ncDeepBrain score was calculated by the first step of iMEGES on the schizophrenia and ASD datasets. To compare the performance of iMEGES for gene prioritization, we generated ROC curves for discriminating the disease genes from the non-disease genes on the schizophrenia dataset and calculated AUC scores. The classification AUC value of gene prioritization of iMEGES is 57% (see Fig. 4 (a)) and 58% (see Fig. 4 (b)) for schizophrenia and ASD datasets respectively. Gene prioritization thus provides useful information to identify disease genes. However, the relatively low AUC values suggest that additional improvements in gene prioritization is needed.

## Discussion

Mental disorders represent significant social and economic toll to the society, and as a group is one of the leading causes of disability worldwide. High-throughput genomic sequencing techniques have enabled the detection of many genetic variants that may contribute to the development of mental disorders. However, the genetic complexity and heterogeneity of mental disorders make the identification and interpretation of genetic variants or genes difficult challenges. In this study, we designed a practical tool, iMEGES, for prioritizing genetic variants and genes that may be associated with specific clinical phenotypes in personal genomes from patients affected with mental disorders. This tool uses a deep learning framework to prioritize variants detected in a personal genome, and thus can enable the identification of specific variants known to be associated with mental disorders, but also help detect novel variants in mental disorders. Further, based on various prediction scores of variants in a personal genome, the second deep learning step in iMEGES is used to prioritize genes associated with specific observed clinical phenotypes in a patient. The top-rank genes are more likely to be disease-relevant genes which might influence susceptibility to mental disorders for a specific patient. The personalized analysis of variants and genes helps identify potential targets, so that the treatment would be more efficient and effective. To the best of our knowledge, there is not such tools available for mental disease for similar purposes. Meanwhile, iMEGES only requires the patient’s genomic mutation data in VCF format (optionally, the detailed clinical phenotypic presentations) and manages all data preprocessing steps for users in an automated fashion, which facilitates researchers to gather a list of prioritized variants and genes easily.

Despite these unique advantages, as one of the first tools for comprehensive prioritization of variants and genes for mental diseases, iMEGES has several limitations which can be addressed in our future development of the tool. First, it is challenging to obtain large-scale high quality data for training statistical models. Since deep learning model was used in iMEGES for classifying the mental disease related mutations and neutral mutations, a large number of high quality data would be helpful. Unfortunately, due to the paucity of data, some of our procedures must rely on imputed GWAS data, and many such hits represent proxy markers rather than true causal variants, making the model less reliable than ideal. Secondly, to use information from the non-coding variants, we associated each non-coding variant to its closest gene in the genome. This strategy may work well for promotors, which explain a fraction of the variations in RNA expression, but for other types of regulatory elements, this strategy may be less optimal. These limitations would be addressed in future, and we expect that iMEGES will be a powerful tool to bridge the gap between the increasing amount of genetic data on patients and the comprehension of the functional impacts of genetic variants in mental diseases.

## Conclusions

We developed a computational tool, iMEGES, for the prioritization of variants and genes that are relevant for mental disorders based on whole genome sequencing data of individual patients. The method can also work as a general approach to integrate additional omics information into the same framework for continuous improvements in identifying disease candidate genes from population-level data. iMEGES prioritizes non-coding variants using ncDeepBrain score, and then prioritizes genes with tissue-specific and phenotype-specific information, and generates prioritize gene scores for mental disorders. We hope that iMEGES can complement existing computational approaches that are not disease-specific, and address the challenge of more sensitive and specific detection of susceptibility variants and genes in personal genomes for mental disorders.

## Additional file

Additional file 1: Supplementary figures for more performance evaluation of iMEGES, and supplementary tables for datasets used, missing values and correlation matrix of different scores for variant prioritization. (DOCX 3608 kb)

## Abbreviations

ChIP: Chromatin immunoprecipitation
CNVs: Copy number variations
DNA: Deoxyribonucleic acid
eQTLs: Expression quantitative trait loci
GTEx: Genotype-tissue expression
nsSNVs: Non-synonymous single-nucleotide variants
QTLs: Quantitative trait loci
SNPs: Single nucleotide polymorphisms
SNVs: Single nucleotide variants
TF: Transcription factor
TSS: Transcription start site
