# Supplementary material for: iMEGES: integrated mental-disorder GEnome score by deep neural network for prioritizing the susceptibility genes for mental disorders in personal genomes
Source: BMC Bioinformatics. 2018 Dec 28;19(Suppl 17):501. doi: 10.1186/s12859-018-2469-7 (PMC6309067; doi:10.1186/s12859-018-2469-7)
Supplement: Supplementary file 1 — Supplementary figures for more performance evaluation of iMEGES, and supplementary tables for datasets used, missing values and correlation matrix of different scores for variant prioritization. (DOCX 3608 kb) [file 12859_2018_2469_MOESM1_ESM.docx]

Supplementary Materials
Table S1. The source and description of compiled algorithms, scores and datasets.

| **Score Name** | **Source Link and Score Description** |
| --- | --- |
| [CADD_CScore](http://www.nature.com/ng/journal/v46/n3/full/ng.2892.html) | <http://krishna.gs.washington.edu/download/CADD/v1.1/whole_genome_SNVs.tsv.gz>  Combined Annotation Dependent Depletion (CADD) is a framework that integrates multiple annotations into one metric by contrasting variants that survived natural selection with simulated mutations. |
| [DANN](http://bioinformatics.oxfordjournals.org/content/31/5/761) | <https://cbcl.ics.uci.edu/public_data/DANN/data/DANN_whole_genome_SNVs.tsv.bgz>  DANN uses the same feature set and training data as CADD to train a deep neural network (DNN). DNNs can capture nonlinear relationships among features and are better suited than SVMs for problems with a large number of samples and features. |
| EIGEN | http://www.columbia.edu/~ii2135/eigen.html  EIGEN is unsupervised machine learning score for non-coding variants. |
| [GWAVA_Region](http://www.nature.com/nmeth/journal/v11/n3/abs/nmeth.2832.html),  [GWAVA_TSS](http://www.nature.com/nmeth/journal/v11/n3/abs/nmeth.2832.html),  [GWAVA_Unmatched](http://www.nature.com/nmeth/journal/v11/n3/abs/nmeth.2832.html) | <ftp://ftp.sanger.ac.uk/pub/resources/software/gwava/v1.0/annotated/gwava_db_csv.tgz>  GWAVA uses the random forest algorithm to build three classifiers using all available annotations to discriminate between the disease variants and variants from each of the three control sets. This control set first was composed of all 1KG variants in the 1 kb surrounding each of the HGMD variants for GWAVA_Region. GWAVA_TSS score uses control set matched for distance to the nearest TSS genome-wide. GWAVA_Unmatched score uses control set constructed from a random selection of SNVs from across the genome in order to sample overall background. |
| FATHMM-MKL | <http://fathmm.biocompute.org.uk/database/fathmm-MKL_Current.tab.gz>  FATHMM-MKL uses MKL classifier to predict the functional consequences of both coding and non-coding sequence variants from various genomic annotations and weights the significance of each component annotation source. |
| ANNOVAR | <http://annovar.openbioinformatics.org/en/latest/user-guide/download/>  ANNOVAR is used for annotations. |
| PHENOLYZER | <http://phenolyzer.wglab.org/>  PHENOLYZER provides scores for genes based on phenotype information as input terms |
| RVIS | <http://genic-intolerance.org/>  RVIS (Residual Variation Intolerance Score) is gene score based on intolerance to mutations. |
| GTEx | <https://www.gtexportal.org/home/>  GTEx provides the gene expression score in various tissues. |
| PsychENCODE | <https://www.synapse.org//#!Synapse:syn4921369/wiki/235539>  We downloaded the enhancer/promoter data from PsychENCODE. |
| CommonMind | <https://www.synapse.org//#!Synapse:syn2759792/wiki/69613>  We downloaded the known eQTLs data from CommonMind database. |

**Table S2:** Missing values for training and testing datasets for variant prioritization.

| **Dataset** | **Training dataset 1 (%)** | **Training dataset 2 (%)** | **Training dataset 3 (%)** | **Training dataset 4 (%)** | **Training dataset 5 (%)** | **Testing dataset 1 (%)** | **Testing dataset 2 (%)** | **Testing dataset 3 (%)** | **Testing dataset 4 (%)** |
| --- | --- | --- | --- | --- | --- | --- | --- | --- | --- |
| EIGEN | 1.2 | 2.23 | 1.05 | 0.91 | 2.18 | 1.46 | 0 | 1.29 | 0 |
| CADD | 0.25 | 1.08 | 0 | 0 | 0 | 0 | 0 | 0 | 5.03 |
| DANN | 0.26 | 1.09 | 0 | 0 | 0 | 0 | 0 | 0 | 0 |
| GWAVA | 0.27 | 1.09 | 0 | 0 | 0 | 0 | 0 | 0 | 0.20 |

**Table S3:** Correlation matrix of the variables in the training dataset 1.

|  | **EIGEN** | **CADD** | **DANN** | **GWAVA** | **FATHMM** | **GNOMAD** | **eQTLs** | **H3K27Ac** | **H3K27me3** | **H3K4me1** | **H3K4me3** | **Labels** |
| --- | --- | --- | --- | --- | --- | --- | --- | --- | --- | --- | --- | --- |
| EIGEN | 1 | 0.60 | 0.36 | 0.31 | 0.48 | -0.07 | 0.00 | 0.20 | 0.05 | 0.14 | 0.33 | 0.06 |
| CADD | 0.60 | 1 | -0.22 | 0.24 | -0.06 | 0.01 | 0.05 | 0.03 | 0.08 | 0.06 | 0.06 | 0.04 |
| DANN | 0.36 | -0.22 | 1 | 0.18 | 0.50 | -0.12 | -0.04 | 0.16 | -0.00 | 0.10 | 0.23 | 0.04 |
| GWAVA | 0.31 | 0.24 | 0.18 | 1 | 0.20 | -0.03 | 0.01 | 0.23 | 0.12 | 0.21 | 0.26 | 0.07 |
| FATHMM | 0.48 | -0.06 | 0.50 | 0.20 | 1 | -0.11 | -0.01 | 0.1 | 0.01 | 0.07 | 0.27 | 0.04 |
| GNOMAD | -0.07 | 0.01 | -0.12 | -0.03 | -0.1 | 1 | 0.00 | -0.00 | 0.00 | -0.00 | -0.02 | 0.04 |
| eQTLs | 0.00 | 0.05 | -0.04 | 0.01 | -0.01 | 0.00 | 1 | -0.01 | 0.02 | 0.00 | -0.01 | -0.00 |
| H3K27Ac | 0.20 | 0.03 | 0.16 | 0.23 | 0.15 | -0.00 | -0.01 | 1 | 0.26 | 0.30 | 0.36 | 0.02 |
| H3K27me3 | 0.05 | 0.00 | -0.00 | 0.12 | 0.01 | 0.00 | 0.02 | 0.26 | 1 | -0.04 | 0.026 | -0.01 |
| H3K4me1 | 0.10 | 0.06 | 0.10 | 0.20 | 0.00 | -0.00 | 0.00 | 0.30 | -0.04 | 1 | 0.40 | 0.03 |
| H3K4me3 | 0.30 | 0.00 | 0.23 | 0.26 | 0.27 | -0.02 | -0.01 | 0.36 | 0.02 | 0.40 | 1 | 0.04 |
| Labels | 0.00 | 0.04 | 0.04 | 0.07 | 0.40 | 0.04 | -0.00 | 0.02 | -0.01 | 0.03 | 0.04 | 1 |


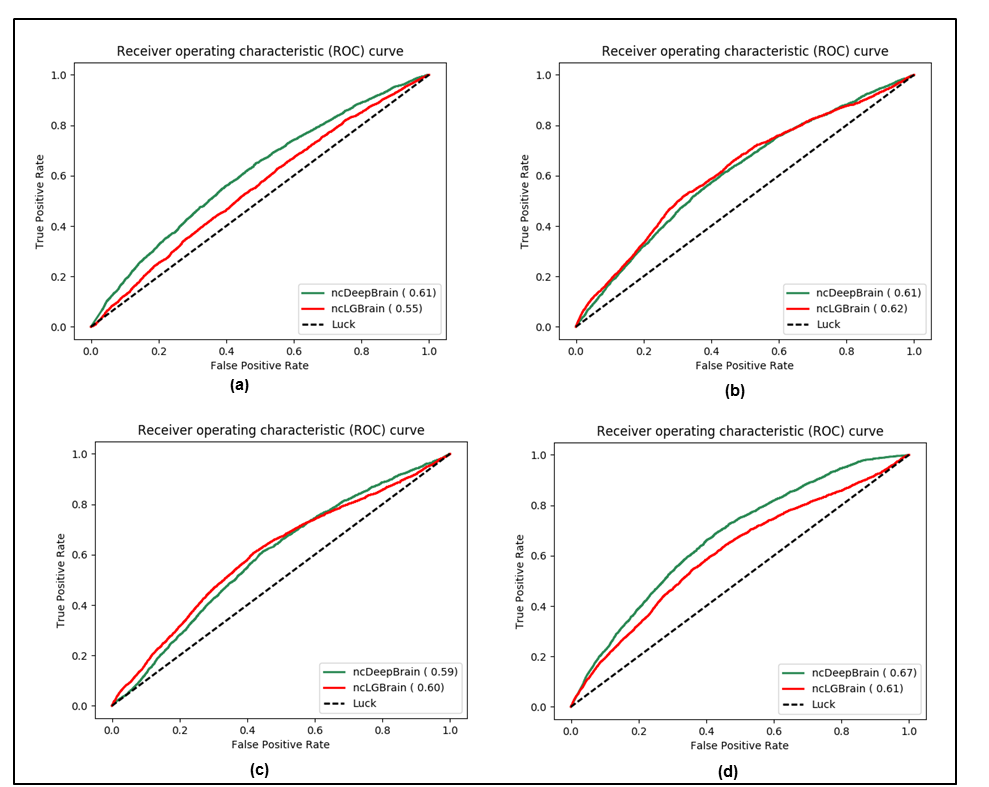


Figure S1. The performance of variant prioritization of iMEGES for non-coding variants. These plots illustrated the performance of ncDeepBrain (a deep learning model) and ncLGBrain (logistic regression) scores by the ROC curve and AUC score. Higher AUC score indicates the better performance power of discriminating the disease variants from the common variants. (a) the performance of ncDeepBrain and ncLGBrain for training dataset 1 and testing dataset 1, (b) the performance of ncDeepBrain and ncLGBrain for training dataset 2 and testing dataset 1, (c) the performance of ncDeepBrain and ncLGBrain scores for training dataset 3 and testing dataset 1 and (d) the performance of ncDeepBrain and ncLGBrain scores for training dataset 4 and testing dataset 1.


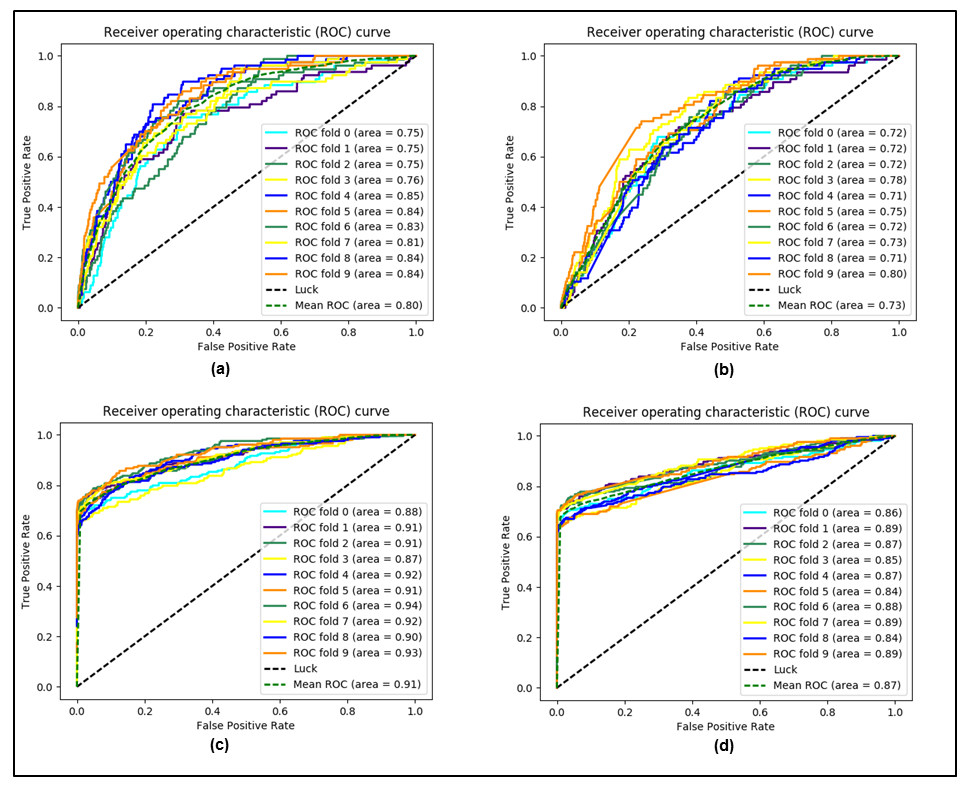


Figure S2. The performance of variant prioritization of iMEGES for non-coding variants. These plots illustrated the performance of ncDeepBrain score by the ROC curve and AUC score based on 10-fold cross-validation. Higher AUC score indicates better performance of discriminating disease variants from normal variants. (a) ncDeepBrain score and (b) the combined non-coding scores such as EIGEN, CADD, DANN, GWAVA-unmatched and FATHMM of training dataset 1. (c) the ncDeepBrain score and (d) the combined non-coding scores such as EIGEN, CADD, DANN, GWAVA-unmatched and FATHMM of training dataset 2.


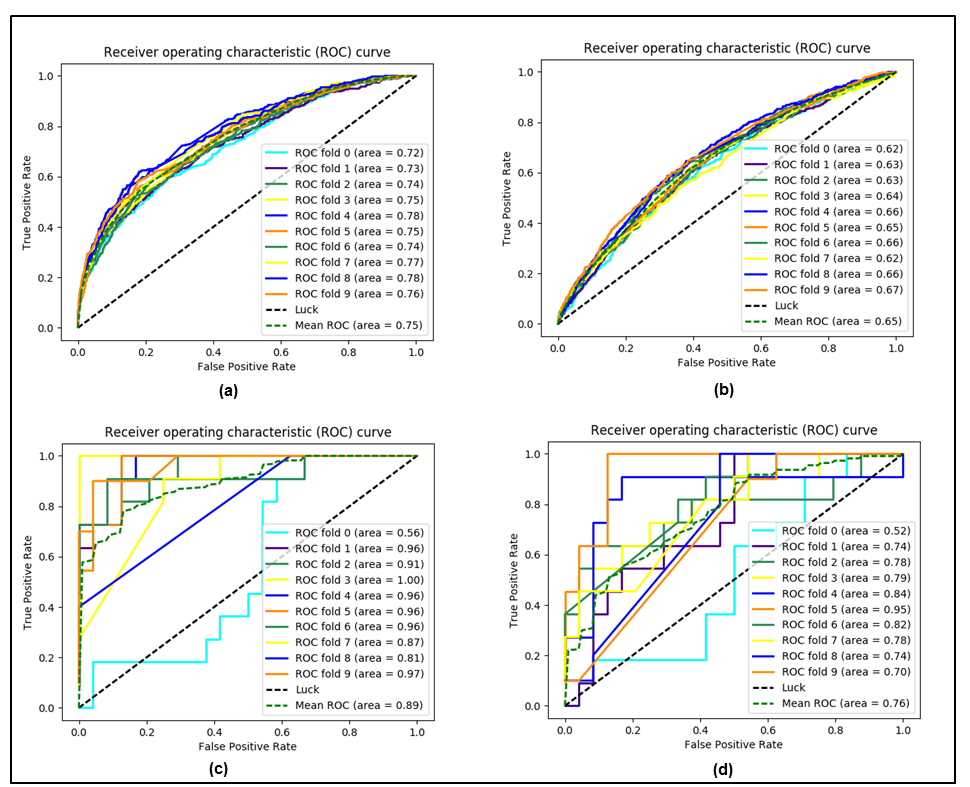


Figure S3. The performance of variant prioritization of iMEGES for non-coding variants. These plots illustrated the performance of ncDeepBrain score by the ROC curve and AUC score based on 10-fold cross-validation. Higher AUC score indicates better performance of discriminating disease variants from normal variants. (a) ncDeepBrain score and (b) the combined non-coding scores such as EIGEN, CADD, DANN, GWAVA-unmatched and FATHMM of testing dataset 1. (c) the ncDeepBrain score and (d) the combined non-coding scores such as EIGEN, CADD, DANN, GWAVA-unmatched and FATHMM of testing dataset 3.


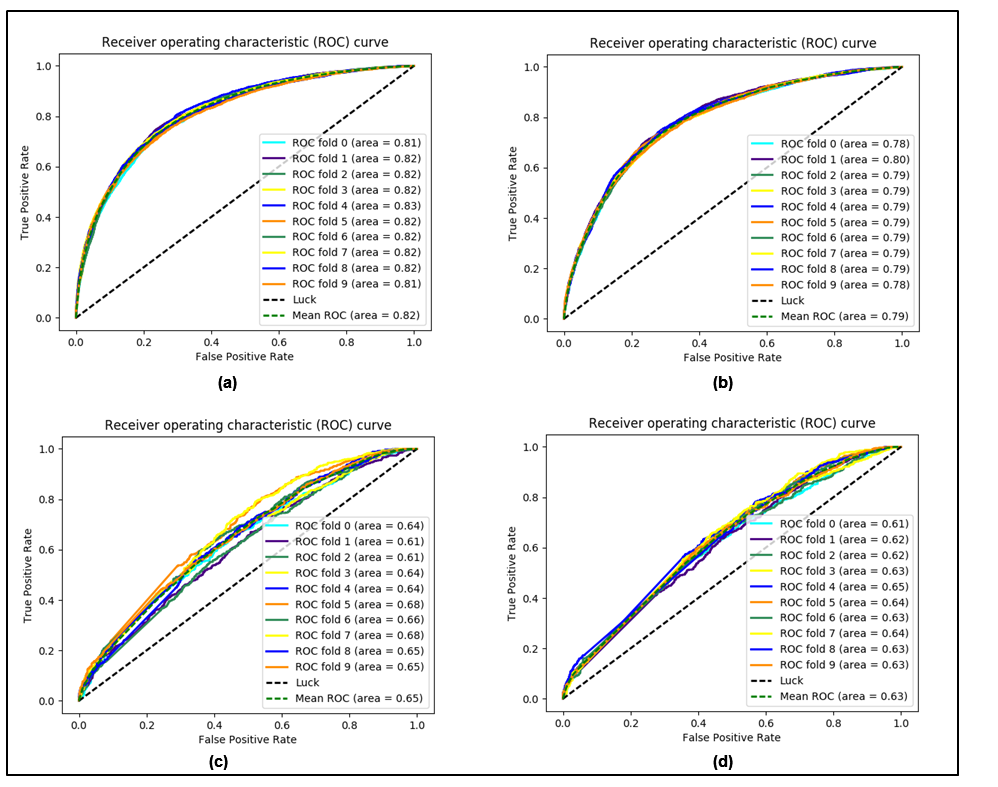


Figure S4. The performance of variant prioritization of iMEGES for non-coding variants. These plots illustrated the performance of ncDeepBrain score by the ROC curve and AUC score based on 10-fold cross-validation. Higher AUC score indicates better performance of discriminating disease variants from normal variants. (a) ncDeepBrain score and (b) the combined non-coding scores such as EIGEN, CADD, DANN, GWAVA-unmatched and FATHMM of training dataset 3. (c) the ncDeepBrain score and (d) the combined non-coding scores such as EIGEN, CADD, DANN, GWAVA-unmatched and FATHMM of testing dataset 2.


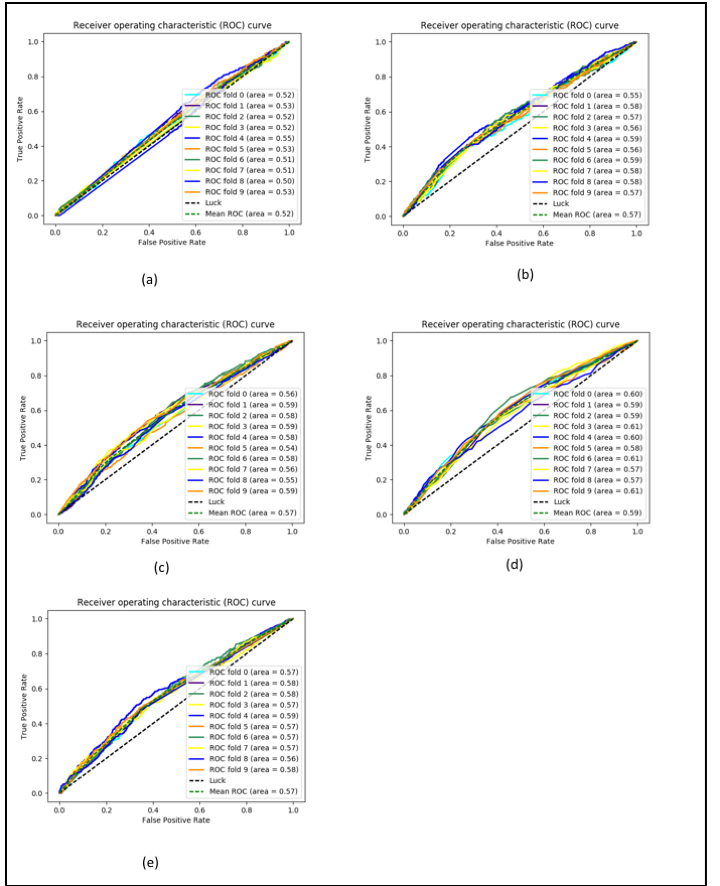


Figure S5. The performances achieved by the EIGEN score (a), the CADD score (b), the DANN score (c), the GWAVA score (d) and the FATHMM score (e) on training dataset 1.
